# Integrating Australian Native Foods for a More Sustainable Food System: A Qualitative Co-Design Study with Aboriginal Communities

**DOI:** 10.3390/ijerph22040646

**Published:** 2025-04-19

**Authors:** Carla Vanessa Alves Lopes, John Hunter, Renee Cawthorne, Shirley Gilbert, Ayoola Shogunle, Cassandra Ebsworth, Mike Bartlett, Rimante Ronto, Seema Mihrshahi

**Affiliations:** 1 Department of Health Sciences, Faculty of Medicine, Health and Human Sciences, Macquarie University, Macquarie Park, Sydney, NSW 2109, Australia; 2 School of Natural Sciences, Macquarie University, Macquarie Park, Sydney, NSW 2109, Australia; 3 Murama Cultural and Healing Centre, Sydney, NSW 2127, Australia; 4 School of Education, Western Sydney University, Sydney, NSW 2751, Australia; 5 Baabayn Aboriginal Corporation, Sydney, NSW 2770, Australia; 6 Sydney Olympic Park, Sydney, NSW 2127, Australia

**Keywords:** native food, Aboriginal, co-design, Indigenous food systems, nutrition, sustainability, health promotion

## Abstract

(1) Background: Integrating native foods into food systems has shown promising benefits for health, the environment, and the revitalisation of Aboriginal culture. This study aims to explore the benefits, facilitators, and barriers of integrating Australian native foods into the current food system and how traditional knowledge around these foods can be revitalised in a culturally safe way. (2) Methods: This qualitative co-design study involved the following four phases: (I) Relationship building with the communities and cultural training for the research team; (II) Establishment of the Aboriginal Reference Group (ARG) for community involvement and governance; (III) Data collection through interviews and focus groups with participants from two urban Aboriginal communities in Sydney and experts in native foods; and (IV) Collaborative data analysis using both deductive and inductive thematic analysis. (3) Results: We interviewed 22 participants who acknowledged the nutritional, health, cultural, environmental, and economic benefits of Australian native foods. They strongly identified the impact of colonisation and imposed Western culture as root barriers impacting other barriers at the structural, socioeconomic, social, and environmental levels. Participants aspire to achieve food security and sovereignty in a more sustainable food system including native foods. To achieve their aspirations, a framework based on Aboriginal values and principles was developed to guide multicomponent initiatives using native foods. (4) Conclusions: A compassionate food model based on emancipatory community-based and land-based education is essential, connecting ancient and contemporary knowledge to transform the food system. Future research should focus on implementing and evaluating the multicomponent interventions suggested by the participants.

## 1. Introduction

Food choices and systems play a major role in shaping environmental and health outcomes, making it essential to adopt changes aligned with global sustainability goals to face three interlinked crises: obesity, malnutrition, and climate change [1,2,3]. To reduce diet-related chronic diseases and minimise environmental impacts, research emphasises the importance of decreasing the intake of animal-derived food while increasing plant-based intake [4,5,6,7], along with the integration of native foods and traditional food knowledge [8,9,10]. In addition to dietary shifts, health is influenced by a range of factors, including the environment, income, education, and access to food and health services [11]. For First Nation Peoples (Aboriginal and Torres Strait Islander people), their cultural identity, connection to the land, family and kinship, language, and traditional knowledge are also key determinants of health. Understanding and addressing these unique factors is essential for effective health interventions [12].

Before colonisation, Aboriginal people in Australia thrived within an environment that supported their health through cultural and healthy dietary practices. Aboriginal diets were diverse, minimally processed, sustainable, and closely tied to their cultural practices and the natural environment [13,14]. First Nations Peoples consumed a wide variety of foods, including numerous seeds, nuts, more than 150 types of tubers and roots, and over 300 different fruits and vegetables. In coastal communities, such as those in New South Wales, marine resources were a substantial part of their diet [15]. Additionally, in southeastern Australia—the area from Brisbane to Adelaide—there were between 90 and 100 different language groups, each with its own food sources, including warrigal greens, muntries, various lilies, wattleseeds, wild bananas, native cherry, and native yams [16]. These diets were rich in nutrients and diverse food sources, contributing significantly to the health and well-being of these communities [17]. Research indicates that many native foods in Australia are denser in nutrients than comparable “Western” foods [18]. For example, some Australian native rice and some acacia seeds, such as *Acacia bilimekii*, contain up to 35.5% protein [19,20,21,22] and high fibre content [20,23,24]. Additionally, some Australian native foods are rich in micronutrients, including zinc, iron, magnesium, calcium, potassium, and vitamins A and C [18]. For instance, the Kakadu plum is the richest known source of vitamin C, containing 7000 mg per 100 g [23], and Eucalyptus seeds (*Eucalyptus pachyphylla* F. Muell) are an excellent source of magnesium, surpassing even sesame seeds [25].

The nutritional and health benefits of Australian native plant-based foods are well-documented [18]. High fibre content, enzyme inhibitors that slow carbohydrate digestion, and the presence of phenolic compounds associated with antidiabetic properties make certain native foods, like Cheeky yam (*D. bulbifera*), blackbean seed (*C. australe*), and wattle seed (*Acacia aneura*), suitable for diabetic-friendly diets [14,26]. Additionally, phenolic compounds such as gallic acid, catechins, and quercetin, along with vitamin C, contribute to the anticancer properties of native foods like Lilly Pilly, Kakadu plum, and Davidson’s plum [23,27,28]. Other native foods offer antimicrobial and antifungal benefits, such as green plum (*Buchanania obovata*) [29], as well as antioxidant and anti-inflammatory properties found in *Carpobrotus rossii* (pigface) [30] and native pepperberry [31]. Aboriginal diets also supported emotional well-being [32] and food security [33], with some researchers asserting that First Nation Peoples were emotionally, physically, and socially healthier than many Europeans of that era [34].

In addition to their nutritional and health benefits, studies highlight the positive environmental impacts of Australian plant-based native foods and their potential to address climate change while fostering more sustainable food systems [18]. Traditional Aboriginal food practices, including methods like controlled burning and seasonal calendars, are increasingly recognised for their environmental benefits, such as enhancing plant, water, soil, and animal health [13,35,36,37,38,39,40]. Furthermore, some Australian native foods are highly resilient, exhibiting tolerance to heat [19,41], salinity, and drought [19,42,43], and certain species also contribute to carbon sequestration and water flow regulation [44]. Aboriginal communities also hold a profound spiritual connection with native foods, where interaction with their lands and foods represents an expression of their identity and connection with their ancestors [32,35,39,45,46]. However, with the invasion in 1788, land occupation by settlers led to devastating impacts on Aboriginal culture and diet [47]. Colonisers forcibly removed First Nations children from their families and communities, introduced violence, and brought epidemic diseases that contributed to a tragic genocide in these communities [47]. Europeans also introduced foods such as white flour, tea, sugar, tobacco, and meat, and by the 1890s, rationing became a new form of control [48]. This strategy, called “assimilation by diet”, discouraged traditional practices like gathering and hunting native foods, leading to a gradual dependency on introduced foods and a loss of Aboriginal food practices and knowledge [16]. This was more than a loss of traditional knowledge and skills; it impacted connections to the land and spirituality, as traditional foods and practices are deeply woven into Aboriginal culture [16].

The forced separation from family, community, and cultural identity has had lasting effects, contributing to intergenerational trauma that continues to affect Aboriginal people. This trauma is widely recognised as a key factor in the persistent disadvantage and poor physical and mental health seen in these communities [49]. Intergenerational trauma can be understood as historical traumatic events experienced by one generation that continue to impact the health and well-being of subsequent generations [50]. Several statistics illustrate the effects of this trauma. First Nations children experience maltreatment at a rate seven times higher than non-Indigenous children, and in 2021–2022, First Nations youth were detained at a rate 24 times higher than their non-Indigenous counterparts [12]. Aboriginal and Torres Strait Islander individuals make up approximately 32% of all prisoners in Australia [12]. Employment rates are also significantly lower among First Nations people compared to non-Indigenous Australians (56% vs. 78%), and about 33% of Indigenous Australians live in housing with structural problems such as plumbing issues or wall and floor cracks [12]. These socioeconomic disadvantages directly impact health risk factors and overall epidemiological patterns within Aboriginal communities [12].

Food insecurity is another significant concern, with 22% of Aboriginal and Torres Strait Islander people reporting food insecurity in 2012–2013, compared to just 3.7% of non-Indigenous Australians [51]. This disparity is partly due to low income and the challenge of accessing fresh, affordable food [52]. Additionally, in 2018–2019, over 70% of First Nations People aged 15 and over were classified as overweight or obese, a leading risk factor for chronic diseases such as type 2 diabetes, cancer, and cardiovascular disease [12]. In 2018–19, 7.9% of Aboriginal and Torres Strait Islander (First Nations) people were living with diabetes, with rates nearly three times higher than those of non-Indigenous Australians [53].

To face this situation and heal these communities from intergenerational trauma, the Healing Foundation [54] recommends that the trauma should be understood in the historical and colonisation context; the First Nation Australians have the knowledge and skills to heal from trauma, reconnection to culture and traditions must be part of the healing process, and healing is more effective when designed, developed, and delivered by Aboriginal and Torres Strait Islander people. Research shows that connecting First Nations communities with their culture through collaborative, participatory approaches—particularly culturally grounded interventions designed and implemented by First Nations Peoples—leads to greater success [55]. In a study with young urban Aboriginal people, Murrup–Stewart et al. [56] found that involving youth in cultural activities, such as engaging with Elders, family, and community members, and reconnecting with their culture through arts, weaving, music, dance, time on Country, community gatherings, traditional foods, and ceremonies, is key to improving social and emotional wellbeing. Several studies highlight the importance of traditional and native foods in fostering cultural connections and improving health and well-being among Aboriginal Australians [57,58,59,60,61]. Conversely, these studies also showed that participants experience a sense of loss and face barriers to healthy eating when they are disconnected from Aboriginal traditional food systems [57,58,59,60,61].

Considering the social and cultural importance of these foods as a cultural determinant of health for Aboriginal communities, this co-designed study explored the benefits, facilitators, and barriers of integrating Australian native foods into the current food system and how traditional knowledge around these foods can be revitalised in a culturally safe way. It is important to note that the initial aim was to explore how knowledge about native foods could be ‘exchanged’ effectively. However, as findings emerged, particularly the barriers due to knowledge loss resulting from colonisation, the study’s focus expanded, shifting towards exploring methods for ‘revitalising’ traditional knowledge around these foods as a foundation for knowledge exchange. This shift occurred because we initially assumed that knowledge about native foods was present in the community, but the research revealed that much of this knowledge had been lost, prompting a need to focus on revitalisation before exchange could occur.

## 2. Materials and Methods

### 2.1. Study Design

A qualitative study design was used, including focus groups and individual interviews, to explore the benefits, facilitators, and barriers of integrating Australian native foods into the current food system and how traditional knowledge can be revitalised around these foods in a culturally safe way. This study encompasses the first four phases of a five-phase co-design protocol, which was published in a previous study [62], including (1) Preparation—a Cultural Safety Training to enhance the team’s capacity to work with the Aboriginal communities; (2) Community involvement and governance—a letter of support from the communities acknowledging their wish to participate in the project, a Research Agreement to ensure communities’ interests, expected benefits and ownership, and the Aboriginal Reference Group (ARG) to ensure Aboriginal leadership; (3) Data collection—individual interviews and focus groups were applied; (4) Data analysis and dissemination of the findings; (5) Elaboration and implementation of the case study, including gardening activities, is ongoing with the communities.

### 2.2. Preparation—Training and Building a Trusting Relationship

The research team attended the Manawari Staff Aboriginal Cultural Safety Training, delivered by Macquarie University in August 2022, to enhance its capacity to work respectfully and reciprocally with Aboriginal communities. More details about the training can be found in our previous study [62]. To build the relationship with the communities, the author (CL) participated in various planned activities and integrated them into their routines, beginning in August 2022 and continuing to the present. These activities included gardening, cultural events, and regular community meetings. All activities were guided and facilitated by the Aboriginal chief investigator, who has kinship connections with the Aboriginal peoples of NSW and established links with the two communities.

### 2.3. Participants, Community Involvement, and Governance

The study was conducted in two Aboriginal communities in Greater Western Sydney, New South Wales—the Murama Healing Space, located in Sydney Olympic Park and Baabayn Aboriginal Corporation, located in Mt. Druitt. Both communities were chosen due to their strong desire to revitalise knowledge of the traditional food system around native plant-based foods and the presence of newly established native gardens. A more detailed explanation of the communities can be found elsewhere [62]. After the communities presented a letter of support, formally acknowledging their wish to participate in the project, a Research Agreement was developed. This document included research aims and methods, Aboriginal community interests, aspirations, and expected benefits to the communities. It also included the ownership of copyright and intellectual property management. An Aboriginal Reference Group (ARG) consisting of five members from both communities guided the research process, ensuring Indigenous leadership and cultural appropriateness. More details regarding the roles and scope of the ARG are described in our study protocol [62]. Community members (CM) were invited to participate if aged 18 years and above and identified as Aboriginal. Preference was given to community members in leadership positions and members involved in garden projects. As part of phase two, consultation with key members of the community, which resulted in a Research Agreement, community members requested that the research team also interview experts (E) who work and study Australian native foods to better understand the benefits, barriers, and facilitators and contribute knowledge back to the community. Experts were recruited through community recommendations, the literature, and through attendance at key conferences. Specifically, community members recommended individuals—both Aboriginal and non-Aboriginal—who had academic, professional, or traditional knowledge of Australian native foods. In addition, the research team identified potential experts through relevant conferences and previous literature, including systematic reviews, to help revitalise and contribute knowledge back to the community. The snowball method [63] was used for further recruitment.

### 2.4. Data Collection

A semi-structured questionnaire, approved by the ARG, was used for face-to-face and virtual individual interviews and focus groups. The interview guide included questions about the community’s aspirations for a sustainable food system, experiences with Aboriginal food practices and Australian native plant-based foods, as well as the benefits, facilitators, and barriers of integrating these foods into our current food system. It also explored how knowledge can be revitalised and how these foods can be integrated into our food system in a culturally safe way. The framework by the Food and Agriculture Organisation of the United Nations [64] was used to explain the definitions of sustainable food systems. A yarning approach, the most used method in Aboriginal and Torres Strait Islander qualitative research [65], was used to encourage participants to share their stories regarding food systems and Australian native foods. The interviews and focus groups were conducted in private settings (online or face-to-face), recorded using Microsoft Teams, and transcribed verbatim. The consent form was explained at the beginning of the focus groups and interviews. A total of 22 participants were included through individual interviews (N = 13), and there were two focus groups, one with 2 participants and the other with 7 (N = 2; N = 7). Data were collected until data saturation [66] was achieved, which occurred during the 22nd interview. To protect participant’s privacy and confidentiality, their identities were anonymised. Each participant was provided with their transcripts for review to ensure the accuracy of their statements. The data collection was conducted by two members of the research team (CL and JH) from March 2023 to April 2024.

### 2.5. Data Analysis

NVivo software (Version 20) was used to import and analyse data using deductive and inductive thematic analysis. A collaborative process was used to analyse the data, including two research team members (CL and JH) and one ARG member (RC). The members analysed the data independently, following the five steps recommended by Terry et al. [67]: (1) becoming familiar with the data, (2) creating initial codes, (3) generating initial themes, (4) reviewing the themes, and (5) then, a group discussion with the research team and ARG resolved any discrepancies to elaborate the final themes. Data analysis occurred from June to September 2024.

### 2.6. Ethical Considerations

This study was approved by the ethics committee at Macquarie University [Project ID 12478] and at the Aboriginal Health and Medical Research Council of NSW [Project ID 2164/23]. Participants signed the consent form and received a voucher of AUD 50 for their participation.

## 3. Results

### 3.1. Participants Demographics

Participants consisted of 12 females (54.5%) and 10 males (45.45%). Eleven key informants were selected from the two communities in Sydney (all identified as Aboriginal) and eleven experts with experience with native foods across Australia. Of these experts, six identified as Aboriginal, and five identified as non-Aboriginal but had significant academic, professional, or traditional/family experience with Australian native foods. The expert group comprised individuals from diverse roles, including farmers, a nursery worker, a chef, a native food industry owner, a project officer at Catholic schools, a professor, a researcher, and individuals involved in projects related to native foods such as native garden consultant and bush and land carer.

### 3.2. Themes and Subthemes

**Figure 1:**
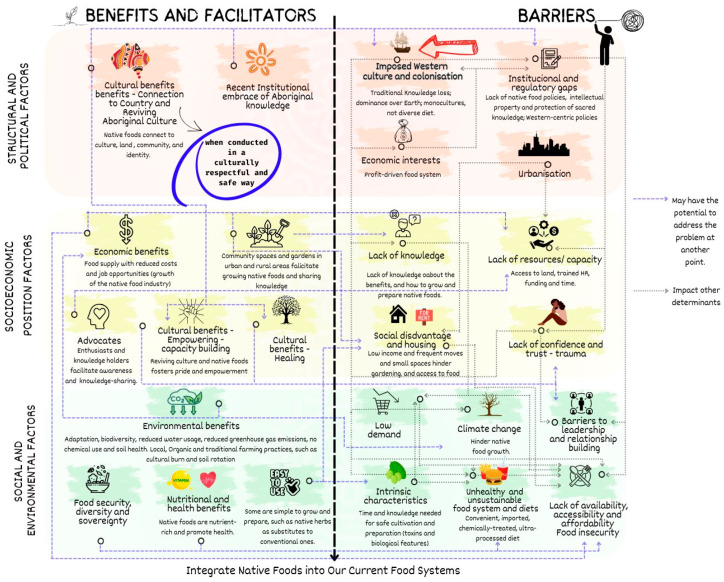
Benefits, facilitators, and barriers of integrating Australian native foods into the current food system and their interconnectedness.

**Figure 2:**
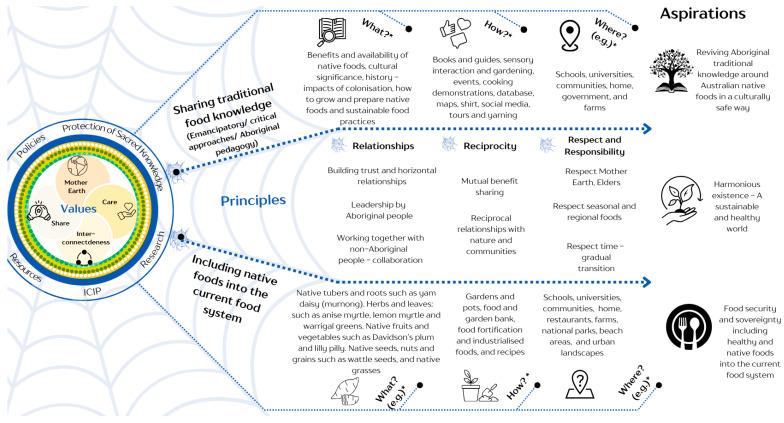
Web of a culturally safe framework to facilitate benefits and sharing opportunities for sustainable food systems and Aboriginal aspirations in the context of integrating Australian native foods into the current food system. * These are examples mentioned by the participants in this study. However, following the values and principles, it is essential to respect the community’s needs, have a culturally safe approach, and respect seasonal and regional foods; ICIP—Indigenous Cultural and Intellectual Property.

Four key themes were identified: (1) benefits and facilitators of integrating Australian native foods into the current food system, (2) barriers to integrating native foods into the current food system, (3) Aboriginal aspirations for the food system, and (4) reviving traditional knowledge and native foods. The emergent themes and subthemes are outlined below and illustrated in Figure 1 and Figure 2. These figures aim to highlight the interconnectedness of the themes and subthemes, analysed through the lens of the three key contextual factors: structural and political, socioeconomic position, and social and environmental conditions. These three contextual factors are aligned with the determinants of health framework as outlined by the World Health Organization (WHO) [68], but some outside the health system. This analysis allows us to examine how broader systemic issues, economic circumstances, and community-level dynamics shape the opportunities and challenges associated with native foods. Themes 3 and 4—aspirations and culturally safe approach—are depicted in Figure 2. Participants’ illustrative quotes are provided in Table A1—Appendix A.

#### 3.2.1. Theme 1: Benefits and Facilitators of Integrating Australian Native Foods into the Current Food System

##### Cultural Benefits

Participants highlighted the cultural benefits of integrating Australian native foods into the Australian food system in a culturally safe way (we explore this theme deeper in Figure 2). A key cultural benefit was the “Connection to Country, which reinforces the deep connection between Aboriginal people and their land and food, promoting a sense of belonging and offering a path to revive Aboriginal culture to sustain cultural heritage, traditions, and identities; as participant E3 described: “*bush tucker means a lot to our reconnection to country*”.

“Empowering Aboriginal communities” emerged as another cultural benefit, as the knowledge and cultivation of native foods can foster community cohesion. “Capacity building” was also noted, as native foods can provide opportunities for skill development, education, and economic growth. Participants also spoke about how engaging with native foods—especially through gardening activities that involve physical connection to the land—can be a powerful form of healing from the intergenerational trauma of colonisation. For example, participant CM5 described how hands-on experiences with plants and soil not only build cultural knowledge but also contribute to emotional and spiritual well-being: *“take people out, and they could do a bit of weeding or, you know, their hands in Mother Earth. But I think if they’d get to know the knowledge or the different plants, they are healing themselves too […] healing [our] way”.*

##### Environmental Benefits

Most participants identified the environmental benefits of Australian native foods. One prominent benefit identified was their “adaptation to a local ecosystem”, which enables these plants to survive in extreme weather conditions such as drought and floods: *“are drought tolerant, they can cope with flooding […] They keep the temperature at least 10 degrees cooler, that’s scientifically proven […] we have lots of companion planting […] can help manage that sort of thing [pests]”* (E4). Participants also emphasised the importance of these foods to support native wildlife and biodiversity, noting that some native animals rely on these plants for survival, as clearly explained by participant E5: *“the main food sources for other animals to help them thrive. [For instance,] he’d always put a little bottlebrush, [because] the honeyeater bird, which was native to the Blue Mountains, used that as their main food source”.* Additionally, Australian native foods can also contribute to climate change mitigation by using less water and chemicals and emitting less greenhouse gas. Soil health enhancement was another environmental benefit mentioned by the participants. Some participants also cited some traditional and sustainable food practices that may benefit the environment, such as cultural burning, the tradition of rotating crops to allow soil recovery, diversity of crops (not based on monocultures), respect for local, regional, and seasonal foods, and the use of companion plants to manage pests.

##### Economic Benefits

The two main economic benefits cited by the participants were “food supply with reduced costs” and “job and market opportunities” as income sources. According to the participants, due to their adaptation to the local ecosystem, Australian native foods grow faster and more easily, which can reduce costs typically incurred by the agricultural industry, such as fertilisers and agrochemicals. Some participants also emphasised the native food industry growth and the importance of these initiatives in creating job opportunities for Aboriginal and non-Aboriginal people. For example, one of the reasons to produce native foods for participant E2 is to create job opportunities for Aboriginal people: “*[…] we’re trying to produce employment for Aboriginal people, we’re trying to improve the prosperity in Aboriginal communities”.*

##### Nutritional and Health Benefits

According to the participants, these foods are nutrient-rich and can contribute to the nutrition of the Australian population, providing vitamins, minerals, fibres, protein, and carbohydrates. Different health benefits emerged from the narratives, including the prevention of chronic diseases such as diabetes, cardiovascular disease, and kidney diseases, as well as the provision of mental health and well-being and a healthy digestive system. Participant E2 highlighted the protein and antioxidant content and the benefits for diabetes prevention: *“Take kangaroo grass for instance, Buru ngalluk—kangaroo grass it had 27% protein as against 11% for wheat. So, by eating our grain, you only need to eat half as much of our bread to get the same effect that you would get from wheaten bread […] fibres are longer and there’s more protein, you’re improving your health […] we suspect that it has three or four times the level of antioxidant that many of those plants […] that’s my recipe for a vanilla Lily or manyang is to eat it fresh. It is such a delicious vegetable you don’t need to do anything to it. It is gorgeous as it is. In the season when they are not as crisp, I fry them in a little bit of butter or oil. You get them to a certain temperature and the sugar in the tuber, which is actually fructose, so it’s diabetic friendly. This is another thing about our food. They are friendly to diabetics. In fact, they inhibit diabetes. That’s why our people never had diabetes because we were eating plants whose sugars were not corrosive to the body”.*

Some interviewees also expressed the importance of these foods to address food security and provide food diversity, as explained by participant E1: *“so much more diversity with native foods because they’re seasonal […] you’ll get different types of bush tomatoes all year round, or you’ll get different types of beans or different types of citrus, and they’ll be in these short seasonal things because that’s when they grow”*, and participant E5: *“the plants that grow in this environment naturally produce the vitamins and minerals that I feel like are relevant to this sort of environment as well. So, I do feel like anyone would be able to achieve food security in that way”.*

##### Facilitators

Despite many structural barriers mentioned by the participants, including lack of institutional support, one participant acknowledged the recent institutional embrace of Aboriginal knowledge compared to some decades ago: “*for example, the Soil Conservation Society had shifted dramatically to think about Aboriginal ways of knowing country, Aboriginal ways of thinking about sustainable practices*” (CM11). Some participants stated that community spaces and gardens are facilitators of sharing food and knowledge about the environment, culture, and bush foods. Participants also noted that advocates can facilitate the sharing of knowledge about these plants. Participant E11 included teachers as potential advocates: *“there are some really strong literacy teachers there who are thirsty about Aboriginal culture, thirsty for Aboriginal culture and respectful and really go out of their way to integrate Aboriginal perspectives and acknowledge it”.*

Some participants considered that some native plants are easy to grow and prepare, which could be a facilitator to include these foods into our food system: *“how easy it is to grow […] how easy it is to prepare […] it’s simple. It doesn’t have to be hard […] there’s equivalent for every flavour […] we’re not playing to our strengths because we’re not using what’s around us that grows here with very little care”* (E4); *“there’s a ginger plant we have an added, and we call it red back ginger […] It’s really easy to grow”* (E9).

#### 3.2.2. Theme 2: Barriers to Integrating Australian Native Foods into the Current Food System

##### Impact of Imposed Western Culture and Colonisation

The impact of imposed Western culture and colonisation was a strong barrier mentioned by most participants that impacted other barriers at all levels, structural, socioeconomic, social, and environmental, as shown in Figure 1. The imposed culture shaped the food and the educational system, and this was one of the main barriers to integrating native foods into the Australian food system and diet. Participants highlighted that the genocide of Aboriginal people due to colonisation significantly contributed to the loss of knowledge regarding Australian native foods and the loss of access to land: *“[…] since colonisation there’s been a whole system by the government to eradicate […] have genocide to our people and that has led to a lot of knowledge loss”* (E1). They described the European system’s belief in human domination over Earth for profit, leading to a commercial food system that excludes traditional practices and is characterised by a lack of diversity, reliance on monocultures and imported crops, extensive use of chemicals, and consequent environment degradation, including problems with seed germination and supply. Like other plants, Australian native flora is also affected by climate change. Participants mentioned that the environmental degradation caused by agribusiness and extreme weather events, such as drought and floods, are significant barriers to growing native plants, as explained by participant E9: *“They’re also heavily affected by rain and flood and things like that. El Nino definitely would have a big effect on a lot of the drought, the drought hardy plants […] a lot of the plants are affected by flood”.*

Additionally, participants noted the predominance of a male-dominated industrial agriculture sector and an education system focused primarily on formal degrees, which represent a major structural barrier to introducing native foods, traditional knowledge, and sustainable practices into the Australian food system, as explained by participant E4: *“We have an oral history. We don’t have a degree. I can’t tell you botanical names, but I can tell you how we learn in our culture”.*

The current convenient, imported, ultra-processed, and cheaper food resulting in inadequate diet habits, influenced by the imposed Western culture and policies based on agribusiness, was noted by the participants as a barrier: *“part of the challenge for us globally is to think about food differently, and by that I mean that fast foods and Western notions of how much we eat everyday need to shift significantly […] we eat much more than our body requires […] we also then rely on the three major food chains as our only source […] we just keep over-planting with things we like to eat rather than things that will grow well in this environment”* (CM11). Participant CM1 discussed the influence of big food corporations on our diet habits: *“[…] being introduced to KFC, Hungry Jacks, Woolies, Coles, Aldi, very fast pace […] it’s just a convenience of not looking after things [plants] and just going to Woolies or Coles or Mac Donalds”* (CM1). On the other hand, participant E7 mentioned that traditional wild harvest would be a barrier to develop the industry: *“we have to get more people engaged in the native food industry in a commercial way in farms and plantations […] you can’t develop an industry based on wild harvest”.*

Some participants also mentioned urbanisation as a significant barrier to integrating native foods into the Australian food system, citing difficulties in accessing land and the lack of shade provided by trees to protect some native species in urban gardens. For participant CM11, buildings are replacing trees in Sydney: *“we keep removing them from the landscape every time we do a new build, particularly out here in Western Sydney, we don’t think about replanting of trees […] there’s so little bushland left, particularly in urban and even semi-rural areas […] where then trying to almost force build native gardens in spaces where they’re out to the elements because there are no shade, there is no shade, there’s little protection”.*

##### Economic Interests and Profit-Driven Barriers

The influence of the capitalist system, particularly through the influence of the food industry and chemical and pharmaceutical companies on food systems, public policies, and academia, complicates efforts to achieve sustainability, and the integration of native foods was also mentioned by the participants. Participant E2 shared his experience seeing the influence of chemical companies on projects involving native foods: *“I’m so frustrated by this industry at the moment because there are so many people who want to adopt Aboriginal plants, but only for profit […] But also capitalism, the chemical companies, the water companies, those who buy water. All of those people are fighting us tooth and nail, well they’re literally fighting us. There’s a war going on in the press against these ideas because there are so many vested interests […] the universities, one of which I belong to, its departments are run by chemical companies, sponsored by chemical companies. How can we hope to be objective under those circumstances? […] I went to a seminar recently, and it was sponsored by chemical company. An alternative food conference sponsored by chemical company and all around you could see that influence. “Oh yeah, let’s have sustainable agriculture. You’re gonna need these chemicals well. And you’ll need more water”. You know the influence, it’s still there”.*

##### Institutional Support, Resources, and Regulatory Gaps

Lack of intellectual property and protection of sacred knowledge was highlighted as a potential barrier for Aboriginal communities to share knowledge about native foods and have the trust to build relationships. Several participants noted the lack of benefit-sharing agreements, which allows the industry to profit from this knowledge without any benefits returning to the community. Participant E6 highlighted that even though Australia is a signatory of some protocols for benefit sharing, the document is not respected in real life: *“Australia doesn’t actually follow or doesn’t have anything in place to kind of make those protections become a reality […] Aboriginal groups are worried as soon as I bring something to the market, what’s stopping a bigger company from the outcompeting them? […] Nagoya Protocol, which is a way to look at or a way to provide recognition to traditional plants and traditional knowledge, in terms of around access and benefit sharing agreements, and Australia is a signatory to the Nagoya Protocol, but it hasn’t been ratified in Australia so, they don’t follow the rules”*. On the other hand, participant E9 mentioned that ownership could be a barrier to commercialise these foods: *“there is an ownership there that can make it very difficult to commercialise”*. The lack of policies, research, and protocols to support the integration of native foods was cited as another barrier. Participants mentioned the resistance from the government to implementing such policies, contrasting this with the greater support provided for Western crops: *“Still very small compared to investment into wheat or rice or citrus, sheep, animals”* (E7).

As a result of lack of policies and urbanisation, lack of right to access to land, lack of funding to programs and projects, skilled human resources to work on farming and producing these foods, and lack of time were statements expressed by the interviewees as barriers regarding capacity. Participant CM9 discussed the lack of access to land: *“Access to the Country as well for First Nations peoples. We don’t all have ownership over our traditional lands. That’s a big issue”.*

##### Lack of Knowledge, Trust, and Confidence—Intergenerational Trauma

One of the most important consequences of colonisation is intergenerational trauma. According to most of the interviewees, lack of knowledge about the history, culture, benefits, and how to grow and prepare native foods, as a consequence of colonisation and imposed Western culture, is one of the main barriers among Aboriginal communities, government, farmers, retailers, and consumers, as mentioned by participant CM7: *“We’ve gotta learn about the plants ourselves because all that knowledge was taken away from us”.* This lack of knowledge leads to low consumer demand for these foods (a social and environmental barrier) and, consequently, low interest from farmers and retailers to produce them. Additionally, the trauma of colonisation has caused a lack of confidence and trust among Aboriginal communities, making it difficult to share knowledge about these plants, speak in public, and build relationships with non-Aboriginal communities, as explained by participant CM3: *“it was a way to eradicate it […] and disempowerment […] they still use the language, but they wouldn’t teach us because they knew that the government would be after their kids. A lot of it is all lost”.*

Participants mentioned the leadership of the native food industry by non-Aboriginal people as a barrier, which affects building a trusting relationship with Aboriginal communities: *“I’m so frustrated by this industry at the moment because there are so many people who want to adopt Aboriginal plants, but only for profit, to make themselves into a guru. They don’t want to include Aboriginal people”* (E2). While a non-Aboriginal participant expressed her frustration with the slow process and the difficulty of building a relationship with some communities: *“it requires a lot of patience, time and travel to build relationships with community […] it just is a very slow process and people a lot of the time they give up because it’s just it’s not going to happen in their lifetime […] it’s very frustrating”* (E9).

##### Socioeconomic Disadvantage—Housing and Food Insecurity

Participants highlighted socioeconomic disadvantage and trauma as a significant issue among both Aboriginal and non-Aboriginal communities, which leads to food insecurity (a social and environmental barrier) and limited access to native foods, as well-explained by participant E11: *“a lot of those people are people who come to us because they don’t have food […] food security is going to be an issue […] it wasn’t just Aboriginal families, though we had a lot of South Sea Islander families and Anglo-Saxon families. So, the whole community had high levels of trauma and high levels of social disadvantage. All the things that go with trauma […] [I] Work with a lot of social housing […] people who are renting, they don’t know how long they’re gonna be there”.* Housing insecurity was identified as another barrier to growing native foods, as people feel uncertain about how long they will be living in their current locations.

##### Lack of Availability, Accessibility, and Affordability

The majority of participants recognised that Australian native foods are still not widely available, accessible, and affordable. This situation is influenced by a lack of policies and investment, low demand, a profit-driven system, regionality, and seasonality. As a result, these foods are not produced on a large scale, making them expensive and affecting their stability in the market. Participant CM7 highlighted that: *“There isn’t a lot of places around that they do sell native bush tucker trees and that. They are very expensive to buy the plants to put back in the ground”.*

##### Intrinsic Characteristics

Participants mentioned the intrinsic characteristics of these foods as barriers. Some native foods are hard to grow and prepare, requiring specific knowledge and time. Additionally, some of these foods contain toxic components, necessitating special preparation methods to make them safe to eat. One participant also noted that some people may not find these plants aesthetically pleasing and, therefore, unsuitable for creating a beautiful garden. Participant CM11 talked about how warrigal greens can be considered an unattractive plant, while participant CM9 described murnong as a hard one to grow: *“because it’s hard to propagate [Murnong] […] it’s hard to grow”* (CM9); *“For many people, Warrigal Green is an ugly plant […] you don’t make pretty houses and pretty gardens from some of our bushfoods and tuckers, but is aesthetic the only thing we should be striving for as a global world. Is it more about food and sustainability, rather than this obsession with better houses and gardens”* (CM11).

#### 3.2.3. Theme 3: Aboriginal Aspirations for the Food System

##### Reviving Aboriginal Traditional Knowledge and Foods

Participants strongly supported reviving Aboriginal traditional knowledge of Australian native foods in a culturally safe way. They emphasised the importance of integrating traditional knowledge and culture into the current food system, as mentioned by participant E6: *“I would like to see recognition of traditional knowledge and culture, Aboriginal ways of doing things, integrated into current everyday food systems”.*

##### Food Security and Sovereignty Integrating Native Foods

Participants expressed the need for food security and sovereignty, emphasising the need to incorporate healthier foods and native foods into the Australian food system and diet. They expressed a desire for a food system that ensures access to nutritious and culturally appropriate foods. The inclusion of Australian native foods was seen as a vital component in achieving food security and sovereignty. Participant E4 highlighted the contribution of native food to food security: *“I want food security, and I think sometimes very complex problems can have very simple answers and to me, it makes sense that we grow plants that are local to our areas”.* Participant E2 expressed his desire for sovereignty: *“I wanted to see how the old people utilise that food for two reasons: the health of the Country, but also as a way of demonstrating sovereignty.*

##### Harmonious Existence—A Sustainable and Healthy World

Participants mentioned the need for a harmonious existence, advocating for a healthier world and food system, as claimed by participant E6: *“a transition, I guess, to more sustainable farming practices would be good”*. Many participants highlighted the importance of integrating sustainable methods in food production, recognising that such an approach is crucial for the well-being of the ecosystem, including human beings, animals, and plants: *“We’re trying to create a farm where animals and plants and people can live in some kind of harmony”* (E2). Their comments reflected an awareness and commitment to supporting changes that lead to a more sustainable and resilient food landscape.

#### 3.2.4. Theme 4: Reviving Traditional Knowledge and Native Foods

##### Values—Interconnectedness, Mother Earth, Sharing and Caring

To achieve their aspirations and create a more sustainable food system, some core values were identified in participants’ interviews that must guide policies, research, and methods of sharing knowledge and including native foods into the Australian food system, including respect for Mother Earth, interconnectedness, and sharing and caring.

For Aboriginal people, the heart of the culture is female, and Mother Earth is a supreme being and must be honoured. Participants highlighted the need to honour and protect the natural environment, recognising that the health of our ecosystems is intrinsically linked to the quality and availability of food. Respecting Mother Earth is not just about sustainability but also about fostering a reciprocal relationship with nature, where Earth is seen as a living entity that requires care and protection. Participant E2 delivered a strong speech about this aspiration: “*the very heart of the culture is female […] we cannot abuse the Earth. She is our mother. We only have one […] we must care for the Earth more […] It has to be loving of the Earth […] we’re not the dominant feature. We’re not the most important animal on that farm that day because the most important animal here today is the bandicoot […] Natchatung nunga, which means through the Mother […] it means we all come through the mother. It is to remind people, particularly men, that would come through the mother and our entire responsibility is to Mother Earth […] You cannot treat this country like Europe. You must treat her like herself. She is an individual. She has to be loved”.*

The value of interconnectedness was a recurring subtheme in participants’ discussions, reflecting their belief that all elements of the food system are interrelated and should be approached holistically: *“everyone thinks of us as separate, but we’re one […] we are one, we are all together, because they look after us and we look after them”* (CM1). Participants explained that humans are part of a living ecosystem where everything is connected. Participant E2 emphasised that plants are active spirits, such as our ancestors: *“they are not just plants, they are active in the world, they are active spirits […] like our ancestors”*. Participant CM10 used the metaphor of a spider web to illustrate interconnectedness: *“It’s (native garden) healing, it’s connection, it’s everything […] You’ve got like a centre of a spider web, and it just veers out into different key points on what this Bush Tucker means […] that spider web going back out and healing country, working communication […] web of life and the web of community […] a web of the natural world, and they all are connected”.*

Sharing and caring emerged as fundamental values among participants, reflecting their commitment to building a food system based on mutual support and compassion, as kindly explained by participant E2: *“we must care for the Earth more […] It has to be loving of the Earth […] We have to learn to love”.* In Aboriginal culture, caring for each other and for Mother Earth is fundamental to guiding our existence. Moreover, sharing food and knowledge is seen as a potent way to empower communities, address food security and achieve a more sustainable food system in Australia: *“that everybody’s responsibility is to grow something and this notion of sharing food differently […] this Western way of thinking about “but they’re pinching your mandarins”. I don’t care. I planted them to be shared”* (CM11). These values highlight the importance of community-driven initiatives and the need for policies that support equitable distribution and collective well-being, reinforcing the idea that a food system based on empathy and cooperation is key to achieving sustainability.

##### Principles (4 R’s)—Respect, Reciprocity, Relationships, and Responsibility

Interviewees also highlighted four principles that must guide any policy or project around Aboriginal traditional knowledge and Australian native foods: reciprocity, relationships, respect, and responsibility. Many participants expressed the importance of Aboriginal communities receiving some benefits in return to ensure a reciprocal process, as explained by participant CM9: *“why can’t we move together as a business? I’m here to support you and your business to grow […] Give me something in return […] I’d like to see First Nations peoples grow their own food but then benefit from that economically as well”* (CM9). Building trust and horizontal relationships with communities was also a recurring theme, consistently emphasised by participants as an essential process for empowering Aboriginal communities. Collaborative work between Aboriginal and non-Aboriginal communities was also mentioned by participants as critical to include these foods into the Australian food system: *“You can’t have two people function of boat. You need at least 25 people to function a boat. So, yeah, you need everyone’s input in the community, and not just Aboriginal community, the wider community, you need”* (CM1). However, leadership by Aboriginal people strongly emerged in the participants’ narratives: *“it needs to be led by First Nations people because that’s our traditional knowledge that has been taken from us and we haven’t been given the privilege to learn about it. So, if anyone’s gonna lead this conversation or lead these businesses, it needs to be First Nations people”* (E1). A respectful and responsible approach was also identified by participants as a principle for a culturally safe method. Within this category, participants highlighted the importance of having culturally appropriate materials and language that respect local culture, and seasonal and regional foods. Respecting time and reviving this knowledge and including these foods gradually was also emphasised: *“I think what you need to do is look at their native nurseries and see what they grow […] it will tell you where it grows well, but anything that’s grown locally in abundance, you know it’s gonna grow in that area. But perhaps there’s a plant that’s important to mob that you’d like to get going again […] in a more gradual way […] move slowly and respectfully”* (E4).

##### Resources and Institutional Support

The importance of institutional support and resources was evident throughout the responses. Participants emphasised the need for policies and research guided by the values and principles mentioned before. Access to land, seeds, technology, and funding were underscored by many participants as essential resources for reviving traditional knowledge and integrating native foods into the Australian food system, as supported by participant E3: *“It would be great if the Aboriginal community were given access to land because they know how to use it”*, and participant CM9: *“technology can also assist sometimes as well. It’s not always about doing things in a traditional way but adapting the principles with the available and relevant technology”.* Ensuring intellectual property and protection of sacred knowledge and safeguarding sacred knowledge from exploitation and misuse were also highlighted by respondents: *“there needs to be protection around, you know, these are sacred ingredients, and this isn’t necessarily public knowledge that anyone or any company can go on and profit from”* (E1).

##### Sharing Knowledge Around Native Foods

Feedback from participants suggested a range of topics and methods for sharing traditional knowledge about Australian native foods. The importance of disseminating information on the health, economic, and environmental benefits of native foods was commonly mentioned: *“If you can show them the cost benefits, the minimal inputs, the way they can convert their farming land into something healthier”* (E3). Participants also emphasised the need to revive the cultural significance of these foods and the impacts of colonisation: *“the only way to do it is to teach the history of this country […] understanding how we treat the land”* (E2). To increase demand for these healthy and sustainable foods, interviewees suggested sharing more knowledge on how to grow and prepare native foods, such as recipes, as mentioned by participant CM8: *“[…] you can have about 3 or 4 recipes to start*”. Another key topic that emerged was sustainability and sustainable food systems. Throughout the narratives, participants also mentioned potential materials and ways to share this knowledge, including books, events, sensory interaction and gardening, cooking demonstrations, databases, maps, social media, cultural tours, and yarning. Participant CM5 expressed her point of view about the native garden in the community: *“that’s my vision of this native garden out here…and to have a space to seat and talk […] look at Davidson Plums, and we’ve got Lillie pillies, and all that […] [we want to] get in there to do that storytelling”*. Participant E11 specifically expressed that the Eight Ways of Learning and Teaching, which is an Aboriginal pedagogy, must be considered when planning activities related to sharing native food knowledge: *“you would need to have it in a way of thinking about the eight ways of knowing and teaching […] having those pillars and those eight ways as being our starting points […] they have to understand how to do it and why”.* Sharing knowledge with kids and young people in schools was also frequently mentioned by participants: *“a book to introduce Bush Tucker into the schools”* (CM1).

##### Integrating Native Foods into the Current Australian Food System

Participants often referenced some ways to include Australian native foods into our food system, including some examples of plants and potential places for integration. Gardening and pots, including both native and non-native foods, were frequently mentioned, with suggested locations including schools, homes, and communities. Food banks were another proposed way, with some already existing in some Aboriginal communities, to address food insecurity. Participant E1 suggested gardens and food banks including native foods: *“local produce and people having their own community gardens […] these smaller companies like sending out boxes and stuff that has local fruits and vegetables. I just think it would be really good to have something like that, that’s native as well because there’s so many”.* Some participants suggested food fortification and industrialised foods as potential ways to include these foods into the Australian diet to enhance the nutritional value of commonly consumed foods, as explained by participant E6: *“maybe the best way forward is through like food fortification […] muesli bars or your breakfast cereal rather than having dried apple in your cereal or your muesli bar, what about you could have dried Kakadu Plum, which has a lot more vitamin C. Also, the native grains I am working with would also work well to fortify wheat flour and therefore address nutrient deficiencies that we are seeing”.* Practical and familiar home-cooked recipes, including native foods, were also a popular idea among participants: *“we’ve made Lemon Myrtle biscuit […] I infuse lemon Myrtle within the biscuit base”* (CM10); *“we make bread from our flour that we make from the [native] grains, but we also make lasagna sheets* (E2). Some commonly mentioned native plants were yam daisy (murnong), warrigal greens, lemon myrtle, native grasses, and wattle seeds. However, it is important to highlight that participants are from different parts of Australia, and these suggestions can vary according to their cultural significance and regional availability.

## 4. Discussion

To our knowledge, this study is the first to explore the benefits of Australian native foods and, simultaneously, the factors impacting the integration of these foods into the current food system in order to develop a culturally safe framework to face these barriers. This qualitative study revealed that Australian native foods are linked to many benefits, including healing from past trauma, environmental benefits such as sustainable farming practices, biodiversity, reduced water usage, and nutritional and health benefits such as nutrient-rich antioxidants and diabetes prevention. The impact of Western culture and colonisation, which introduced non-native food systems and disrupted traditional practices, was one of the main barriers cited by the participants to integrating Australian native foods into the current food system. This theme was mentioned as the root of other barriers, such as lack of knowledge and trust, trauma and social disadvantage, lack of institutional support and resources, barriers to building relationships, and unhealthy and unsustainable food systems. The benefits and facilitators mentioned by the participants may have the potential to address the barriers. Moreover, participants aspire for a harmonious food system by reviving Aboriginal traditional knowledge and integrating Australian native foods into the current food system in a culturally safe way to achieve food sovereignty and security in these communities. The approach must be reciprocal and respectful, led by First Nations peoples based on trusting relationships, and guided by values including respect for Mother Earth, interconnectedness, sharing, and caring.

The social, environmental, nutritional, and health benefits cited by the participants align with the previous literature [18]. Studies have shown that some morphological and physiological adaptations make Australian native plants more resilient to extreme conditions, such as root exudates that modulate soil microbial communities, improving nutrient availability and root aeration [69], ephemeral roots, starch storage, and ease of resprouting [70]. These native plants can also contribute to temperature regulation through their symbiotic relationships, such as supporting the growth of beneficial rhizobial bacteria and nutrient availability [71] and promoting beneficial invertebrate populations, which is natural pest control, avoiding the use of chemicals in the soil [72]. Aboriginal cultural burning practices are also recognised as sustainable land management that significantly promotes biodiversity and reduces the fuel load available for more extensive and uncontrolled fires [73,74]. Studies also highlight the potential of Australian native grains to prevent diabetes through their high fibre content and antioxidant properties, controlling glycemic levels [18,75]. Pour et al. 2024 [75] showed that replacing only 10% of conventional wheat flour with native grain flour significantly decreased blood glucose after meals. Studies also demonstrate the significant source of protein in some Australian native foods, which can be a potential option for those seeking a plant-based protein diet, such as native grains containing over 20% protein [76] and the pindan walnut (Terminalia cunninghamii), has been documented to contain approximately 30% protein on a fresh basis [77].

Aligned with the findings from this research, studies in Canada similarly found a pattern of barriers and facilitators affecting Indigenous communities’ access to native foods and healthy eating [78,79,80]. Elliot et al. [78] explored Aboriginal perspectives on traditional food access in Vancouver’s urban setting, highlighting the essential role of native foods in promoting food security for off-reserve Aboriginal populations, now comprising 60% of Canada’s Aboriginal population. Participants identified multiple obstacles to accessing these foods, including colonisation impacts, urbanisation, insufficient government support, loss of traditional knowledge, restricted land access, high living costs, reduced food availability, and climate change [78]. Conversely, they underscored the importance of reviving traditional knowledge, empowering Aboriginal communities, fostering intergenerational connections, advocating for Aboriginal leadership in food policy, and expanding land access to support urban food security [78]. Similarly, Shafiee et al. [79] interviewed urban Indigenous Canadians and reported similar barriers to healthy eating, including limited access to traditional and fresh foods, high costs, urban lifestyles, inadequate government support, and climate change. Home gardening was mentioned as a facilitator [79]. Grann et al. [80] also found that barriers such as historical identity loss, loss of traditional food knowledge, private land ownership, lack of resources, and the expense of traditional foods further hindered Indigenous communities’ access to native foods.

Participants in our study highlighted the economic interests posed by big corporations as a barrier to more sustainable food systems integrating native foods. Similarly, previous studies have analysed and highlighted how agrifood corporations pose significant barriers to sustainable and healthy food systems, primarily due to conflicting interests between public health and corporate profit [81,82,83,84,85,86]. Corporate power within food systems challenges the implementation of sustainable food policies, as economic priorities frequently overshadow environmental and social goals [81,86]. One main obstacle is the political power held by the ultra-processed food industry [82]. For instance, Bene [83] demonstrates the consolidation of power among major “Big Food” corporations in recent years, with the top ten pesticide companies controlling 94% of global sales, six of which also dominate 75% of private-sector crop research. Furthermore, three companies alone control 77% of the farm machinery industry. The lobbying activities of these powerful corporations often involve providing financial incentives to politicians and key decision-makers or actively challenging proposed policies in the media and in court [83], which may explain the ‘silence’ surrounding this issue in several spaces, such as conferences that are intended to address key challenges in food systems [87,88].

Bene highlights the influence of the red meat industry, which produces 304% of the amount recommended by the EAT–Lancet guidelines for sustainable consumption [83]. Lencucha and Thow [85] argue that the neoliberal policy paradigm further strengthens corporate influence over public policy, enabling commercial interests to influence public policy decisions toward profit rather than public health. In Australia, neoliberal policies have prioritised productivist farming by emphasising large-scale, intensive production, weakening local food security and reducing both sustainable practices and agricultural diversity [84]. This free-market ideology has led to the replacement of native foods and traditional agriculture with commodity-based farming focused on monocultures, agrochemical use, and ultra-processed foods [89]. Big Food corporations employ integrated and rapidly evolving marketing techniques aiming to increase the consumption of unhealthy and affordable products [90].

Participants in this study pointed out the lack of protection for Indigenous cultural and intellectual property (ICIP) and Indigenous ecological knowledge (IEK), which has hindered the development of trusting relationships with non-Indigenous people for sharing traditional knowledge. ICIP includes the rights of Aboriginal and Torres Strait Islanders over their cultural heritage, encompassing languages, sacred sites, cultural objects, expressions, and IEK [91]. IEK refers to First Nations’ knowledge about native plants and animals and their associated stories, songs, language, and practices [91].

In the international context, several key instruments support and protect IEK and ICIP. The United Nations Declaration on the Rights of Indigenous Peoples (UNDRIP) [92], the United Nations Convention on Biological Diversity [93], and the Nagoya Protocol [94] are among the most significant instruments. Article 31 of the UNDRIP [92] outlines: “Indigenous peoples have the right to maintain, control, protect and develop their cultural heritage, traditional knowledge and traditional cultural expressions, as well as the manifestations of their sciences, technologies, and cultures, including human and genetic resources, seeds, medicines, knowledge of the properties of fauna and flora, oral traditions, literature, designs, sports and traditional games and visual and performing arts. They also have the right to maintain, control, protect and develop their intellectual property over cultural heritage, traditional knowledge, and traditional cultural expressions”. The Nagoya Protocol, an outcome of the United Nations Convention, emphasises the fair and equitable sharing of benefits (both monetary and non-monetary) from using genetic resources, including native plants [91,94]. The protocol acknowledges the value of IEK and respects Indigenous people’s connection to their Country [91], marking a significant step forward in sustainable development, food security, and public health. These frameworks are intended to facilitate benefit-sharing while recognising the rights of Indigenous and local communities (ILCs) [95,96]. However, the effectiveness of these documents has been debated worldwide, particularly regarding the actual participation of ILCs in decision-making processes and the equitable distribution of benefits [95].

Significant challenges remain in ensuring that the benefits of genetic resources are shared equitably and that the rights of Indigenous peoples are fully recognised and respected [91]. Although Australia signed the Nagoya Protocol in 2012, it has yet to ratify its guidelines [91]. The Australian government has implemented domestic legislation to align with the Nagoya Protocol, which includes the Environment Protection and Biodiversity Conservation Act 1999 (EPBC Act) [97]. However, efforts to implement the Nagoya protocol principles remain fragmented across states [91].

Due to historical exploitation and misappropriation of Indigenous knowledge in Australia, many Aboriginal people avoid sharing their knowledge or entering the bushfood market [91]. For example, global cosmetic companies use Kakadu plum—known for its high vitamin C content—without recognising or benefiting Indigenous IEK and ICIP [91]. Currently, Aboriginal businesses represent less than 15% of Australia’s native food industry, which generates AUD 21 million annually [91]. Challenges for Aboriginal businesses include regulatory compliance, limited Aboriginal leadership, insufficient funding, and the complexity of balancing social and cultural priorities with business development [91]. To achieve a sustainable and culturally respectful Australian native food industry, it must be Aboriginal led [91]. Different stakeholders, including Indigenous communities, research institutions, private sector entities, and government and non-government organisations, must understand and contribute to the protocol’s implementation [94] by aligning policies, legislation, and action plans [98].

Participants in our study identified intrinsic characteristics as barriers to using some native plants, citing toxic compounds and the challenges of growing and preparing these foods. Similar challenges are also noted in the literature [18,99,100]. However, while some Australian native foods contain potentially toxic compounds, like oxalates, these can be reduced or removed through proper preparation techniques, which highlights the importance of traditional food practices knowledge in ensuring the safe consumption of these foods [18,99,100]. Climate change, another barrier mentioned by the participants in our study, is also described in the literature [41,101].

Shifting focus from barriers to aspirations, participants in our study expressed strong aspirations to achieve sustainability, food security, and food sovereignty by revitalising Aboriginal traditional knowledge and integrating native foods into the current food system. These aspirations are grounded in core values and principles that together provide a guiding framework for interventions. Similarly, the literature highlights the deep ancestral connection that Indigenous people have to their food system, which links people to each other, to their Creator, and to all forms of life [102]. Values such as respect and protection for Mother Earth, the interconnectedness with land, people, and nature, and a culture of sharing and caring are widely documented across studies worldwide as critical Indigenous principles for a more sustainable and healthier planet [103,104,105,106,107,108,109,110,111,112,113].

Respecting Mother Earth and the feminine involves recognising our interconnectedness—that we are all one, and we are part of nature as living beings [105,106]. This value is closely tied to the principles of relationship, responsibility, and reciprocity, where we honour the living entity Mother Earth, take responsibility for her care, and cultivate a reciprocal relationship with her, bringing mutual benefits [105,106]. Gratani et al. [109] explored the environmental values of an Australian Indigenous community, where participants also emphasised their central beliefs of dependence and interconnectedness with animals, plants, and land. Interconnectedness extends across time, linking the past to the future by fostering a connection with our ancestors through nature [103]. Similarly, Menzies et al. [111] conducted interviews with Indigenous communities across Canada to explore values to create a more sustainable environment. Participants listed eight core values: responsibility to care for the land; respect for the land; reciprocal relationship with her; moderation—taking only what is needed; gratitude for the ‘gift’ (natural resources); humility—humanity’s place within, rather than above, nature; and agency, acknowledging that the land has its own spirit and capacity for self-regulation, resonating with the principle of respecting seasonality and regionality in food systems noted by our participants.

Additionally, and also similar to our results, participants emphasised mindfulness or interconnectedness, and the importance of respectful and reciprocal relationships with each other and with nature, honouring traditional knowledge and Elders [111]. Reciprocity, derived from the Latin word *reciprocus*, meaning ’back and forth’—much like the motion of a boomerang—is essential for transformative sustainability [114]. This concept, brought to life through the wisdom of our Elders, involves circular actions, interactions, and experiences between people and nature that create mutual benefits [114].

Despite historical oppression, the Indigenous values of love, caring, and sharing remain strong within Indigenous communities [104]. McKinley and Walters [104] explain that food sharing and generosity among Indigenous people reflect resilience, cooperation, unity, and social cohesion. According to Gilbert [107], caring and sharing hunter/gatherer lifestyles were common among our ancestors, where individualistic and selfish behaviours were shamed. Caring and sharing behaviours also have physiological benefits, such as reducing stress, and activities like cooking and eating together foster enjoyment and happiness [107]. With the agriculture model, where we started producing more than we could eat, more competitive, controlling, and holding behaviours started creating huge disparities in wealth distribution and social power [107,115]. As people grew wealthier and more powerful, compassion diminished, and sharing was increasingly replaced by accumulation [107].

Building trusting relationships, another principle highlighted by our participants, is also well-documented in the literature. Oster and Lightning [116] highlight the importance of fostering trust with Indigenous communities, given the historical mistrust and exploitation from colonisation. The authors emphasised that building trust requires recognising Indigenous self-determination and leadership through participatory approaches [116]. Key points include engaging with humility, developing reciprocal relationships, and showing genuine emotional and personal involvement [116].

Holistic Indigenous values and principles are essential to achieving ecosystem health and food sovereignty [117]. In line with the extensive literature on food sovereignty [118,119,120,121,122,123,124,125], some participants in our study expressed a strong aspiration to achieve food sovereignty by identifying and addressing historical and social issues, such as the trauma of colonisation, and by reforming oppressive structures, including the education system and corporate and gender imbalances in the food system. Patel highlights how power dynamics within this system often disregard both Mother Earth and the feminine, resulting in disproportionate food insecurity rates among women, who earn 25% less than men in agriculture [121]. Anderson et al. [126] describe this as a global ecocide and feminicide.

The term ‘food sovereignty’ was introduced by La Via Campesina in 1996 and is defined as ‘the right to define their own food system producing culturally and ecologically healthy foods using sustainable methods’ [119,127]. La Via Campesina describes the difference between the dominant food system vs. the food sovereignty model across various levels, emphasising that food should not be used as a weapon, where people’s next meal depends on shifts in the global economy [119]. Key distinctions include (1) production priorities: agro exports vs. food for local markets; (2) concept of food: a commodity vs. a human right; (3) achieved food security: by importing the cheapest foods vs. locally produced; (4) control over natural resources (land, water, etc.): privatised vs. community-controlled; (5) rural credit sources: private banks vs. public sector; (6) monopoly and oligopoly: present vs. not presented; (7) farming technology: monocultures and intensive use of chemicals vs. agroecology (sustainable practices without GMOs or agrochemicals), with farmers and traditional communities as guardians of cultural heritage [119].

Food sovereignty goes beyond food security, incorporating two fundamental Indigenous principles of food and health: relationality and reciprocity [128]. Indigenous food sovereignty (IFS) is defined as ‘the Indigenous right to land, food and the control of food production ensuring culturally, spiritually and ecologically respectful relationships with all—plants, animals, environment and communities’ [127]. Studies have identified key elements for achieving IFS, including community ownership, collaboration, transmission of traditional knowledge, cultivation and consumption of native foods using traditional food practices, and environmental sustainability [102,127,129]. Consistent with our participants’ perceptions, Jernigan et al. [102] found that having access to resources such as land, seeds, traditional agroecology knowledge, and supportive sustainability policies are also critical indicators for building community capacity for health and food sovereignty. From an Indigenous perspective, agroecology is presented as a proposal to revive Indigenous cultures and their sustainable way of producing food in harmony with Mother Earth [123]. It embodies an Indigenous, feminist, anti-colonial, and antipatriarchy ideology [123,124].

Achieving such ambitious reform in the food system requires, as many studies and our participants advocate, more emancipatory pedagogical approaches to face the ideological and structural elements of the current oppressive model [118,120,123,124,125,130]. Most of these approaches are grounded in ‘critical pedagogy’ or ‘pedagogy of the oppressed’, developed by Brazilian educator Paulo Freire [131]. Recognising that food systems have been shaped by exploitation, dispossession, patriarchy, and racism, critical pedagogy offers a dialectical approach in which people begin by understanding their reality. This involves analysing their local communities, connecting these insights to broader national and international structures, and developing creative solutions that address the economic, social, political, and agricultural structures shaping our food system [118]. For Freire, engaging with and listening to communities is essential [131].

Concrete examples of food sovereignty, agroecology, and critical food system education are found in many countries [125]. A prominent example is the Landless Workers’ Movement (MST) in Brazil, the largest agrarian social movement in Latin America [120]. Through land occupations, they pressure the government to redistribute unproductive lands, which they now use for community living and food production [120]. As part of their agrarian reform efforts, MST established an alternative agroecological education model and a seed sovereignty program known as “education of the countryside (educação do campo)” [120]. In this program, high-school students from the agrarian reform settlements and Indigenous communities receive training in agroecology, focusing on sustainable seed use and natural inputs, while learning about the structures that shape our food system and how to transform it [120]. The seed bank organised by the movement is a powerful example of breaking market dependence, which often forces farmers to use hybrid seeds requiring mandatory agrochemicals and fertilisers. Some of these socially organised movement activities have influenced the creation of laws, governmental offices, and national programs [120], which Pimbert [125] argues are essential for sustainable changes.

One participant highlighted the Eight Ways of Learning and Teaching as an approach for revitalising traditional knowledge around Australian native foods. Known as Aboriginal pedagogy, this method places culture at the core of all thinking and learning, using eight ways to share knowledge: community and land links, story sharing, learning maps, non-verbal and non-linear communication, symbols and images, deconstruction, and reconstruction [132]. Storytelling, another method emphasised by participants in our study, is an important component of Indigenous holistic education [133]. It is deeply connected to the Country and is used to validate Indigenous experiences [133]. Poelina et al. [133] used a Kimberley place-based cultural story of Dangaba, illustrating how storytelling enables Country to become the central teacher. This approach, aligned with Freire’s critical pedagogy, opens a dialogue linking cultural knowledge to contemporary issues, fostering environmental awareness and inspiring sustainable transformation to address climate change [133]. Trinidad [130] also suggests a Critical Indigenous Pedagogy of Place (CIPP) to Indigenise community-based work to motivate youth to learn their own story and past.

Implementing these emancipatory approaches, participants suggested some activities that must be led by Aboriginal people to share knowledge and incorporate native foods into the food system, including booklets, cooking workshops, gardening, community events, food banks, food fortification, and media campaigns. Similarly, many studies around the world have shown the potential of multicomponent interventions using native foods to promote health in Indigenous communities [134]. Research demonstrates nutrient intake and food security improvements through interventions combining educational materials and food distribution [135,136,137,138]. A community-led program in India distributing native and non-native foods improved energy, protein, dietary fibre, and iron intake among the participants [135]. Studies show that media campaigns are linked to reducing social discrimination against native foods [139,140], and gardening and other land-based activities may improve mental health and healing from trauma [137,138,141]. Furthermore, participatory and culturally relevant activities, such as engaging Elders to transmit traditional knowledge [137,138,142,143,144] and incorporating Indigenous languages [139,144], foster a sense of belonging, pride, empowerment, food sovereignty, and build trust within communities.

## 5. Strengths and Limitations

This study presents many strengths. First, the participatory approach, guided by the ARG, ensures that the research is culturally safe and remains aligned with community aspirations and international protocols for working with Indigenous communities. Second, our study provides a foundational framework developed with Indigenous communities in Australia, centred on sustainable food systems and Aboriginal aspirations. This framework includes Aboriginal values and principles, as well as practical activities that honour Indigenous knowledge and priorities, being a basis for future initiatives focused on sustainable and culturally relevant practices with native foods in these communities.

Our study also had some limitations. The sample size, focused on these two urban communities and some experts, though rich in depth, may not fully capture the diversity of perceptions within the Aboriginal population across Australia and may limit the generalisability of findings. Finally, while qualitative methods are valuable for capturing detailed narratives, complementary quantitative methods can be applied in future research to assess the broader impacts of these interventions on health and environmental outcomes. We did not collect demographic information about the participants, which may have limited the depth of our analysis and the potential to contextualise the findings more comprehensively.

## 6. Conclusions

This study aimed to explore the benefits, facilitators, and barriers of integrating Australian native foods into the current food system and culturally safe methods for revitalising traditional knowledge around these foods. The impact of colonisation and imposed Western culture was mentioned by the participants as a root of other barriers permeating structural, socioeconomic, social, and environmental levels. Despite these challenges, they acknowledged the social, cultural, environmental, and economic benefits of native foods.

To achieve food security and sovereignty within a more sustainable food system, participants advocated for emancipatory educational approaches. Such approaches should involve multicomponent interventions, including community events, gardening, cooking activities, educational materials, and media campaigns.

Australian native foods have the potential to address both health and environmental issues. However, their integration into food systems must be led by Aboriginal people and grounded in core values and principles. It is our responsibility to respect Mother Earth and recognise the interconnectedness between us and nature and between past and future. Having more reciprocal and love–caring relationships, honoring Elders and their traditional knowledge, as well as respecting the seasonality and regionality of these foods are also essential components in this process. Moving forward, institutional support, resources, such as access to land and protection of sacred knowledge and Indigenous cultural and intellectual property, alongside Aboriginal leadership, are crucial.

As part of our reciprocal relationship with the communities, for a while, we have developed educational materials based on their requests during this co-design process, aiming to support community capacity and revitalise traditional knowledge around native foods. However, for the next steps in phase 5 of our protocol, collaborating with the Aboriginal Reference Group (ARG) will be essential to develop actionable projects grounded in insights from this co-design process. These projects will enable the systematic implementation and evaluation of the proposed initiatives, assessing their impacts on food security, health, and cultural revitalisation. Phase 5 is a crucial stage to refine these interventions and generate evidence that can support broader policy development and community-based strategies for incorporating Australian native foods into the current food systems. For future research, we acknowledge the importance of exploring processing methods—particularly approaches that align with traditional practices and promote minimally processed foods, which are often more culturally appropriate and nutritionally beneficial.

## Data Availability

Due to the historical and ongoing trauma of colonisation and the misuse and exploitation of personal information experienced by Aboriginal and Torres Strait Islanders, the data collected and analysed during this study are not publicly available. One of the conditions described in the consent form was that only summary and thematic data would be published.

## Appendix A. Participants’ Quotes

**Table A1:** Illustrative Participant Quotes.

**Theme 1: Benefits and facilitators of integrating Australian native foods into the current food system**	
**Subthemes**	**Illustrative Quotes**
Cultural benefits	*“our intimacy with the country, our intimacy with those plants. We know them very well and we see the plant as part of us” (E2).* *“we’re having that cultural knowledge, we’re strengthening each other, we’re learning more about the plant and about each other as well. We become a solid unit or stronger community”* (CM10).
Environmental benefits	*“Because it belongs here […] we had all this stuff and we survived in a climate that is hard to survive”* (CM7). *“they will need less fertiliser, less water”* (E7). *“we didn’t have to manage pests. We basically had an environment that sustained itself […] we actually are trying to find ways to utilise what are pests to benefit our environment”* (E3).
Economic benefits	*“maybe six or seven species now, which are very good because of the growth of the native food industry […] Lemon myrtle, native pepper, Davidson Plum, lemon aspen”* (E7).
Nutritional and health benefits	*“It’s all good for your mental health”* (CM8). *“The other challenges I think are just people’s general understanding of how the natural food is low in sodium, low in sugars high in fibres and though all of those are essential properties for better health. Particularly for cardiovascular, circulation, diabetes, renal failure”* (CM11).
Facilitators	*“it’s not just the garden, it’s where the garden is situated and the stories we can tell and share about the river, near salt water and freshwater meat and all of those sorts of things which are very important stories to not just only the story of the bush tucker garden, but also to long term sustainability, but also trade routes that were happening between different communities up and down the Hawkesbury the Nepean and the Parramatta rivers”* (CM11).
**Theme 2: Barriers to integrating native foods into the current food system**	
Impact of imposed Western culture and colonisation	*“A lot of people weren’t allowed to go after the bush tucker”* (CM8). *“European system […] which believes that man shall have dominion over the Earth […] what has introduced Roundup into our system. That is what has brought so many birds and animals to the point of destruction. That is what reduce the whales to an alarmingly small population in the 70s […] That’s what farming is, war. A war against […] the unresponsive earth […] we talk about industrial agriculture, we’re talking about an industry that is dominated 95% by men”* (E2). *“I’ve eaten too much cheese and biscuits this week […] it’s more about this is what we eat now […]Aboriginal people would, you know, would very rarely eat red meat. So it wouldn’t be something that would be on a table which we do now have a big massive steak, potatoes, carrots or baked, you know, all deep fried”* (CM10).
Economic interests and profit-driven barriers	*“Big Pharma were paying the department to run trials. Those trials were implanting things into animals into food chains”* (CM11). *“bigger companies can have, can now produce them, can make it cheaper. We saw this recently with Bayer (who have merged with Monsanto) beginning a research project with xx to select and breed native grain species* (E6).
Institutional support, resources, and regulatory gaps	*“[…] in those little halls of government, it’s hard to get through the door”* (E3). *“the basic research hasn’t been done to upscale these crops”* (E6). *“finding enough good Aboriginal people that can access the funding and then can stick to modern horticultural practices which demands discipline and skills, resources, training, water, land, all of these things”* (E7).
Lack of knowledge, trust and confidence—intergenerational trauma	*“the knowledge is lost and the plants aren’t successful when they’re being grown, because what’s missing is the early connection to the plant and the knowledge that’s coming from elders and from community”* (E4). *“a barrier is, you know a whole brand-new food that people don’t know what to do with it, they may not have recipes or cooking ways […] It’s hard for people to change their habits in that way and test a whole brand new food that they may know nothing about”* (E5). *“You don’t go and invest into a broad-scale horticulture to propagate thousands and millions of plants and hope you’re going to sell them when people do not know what to do with them”* (E7). *“Some are intimidated by people that are educated or even being confident to be, to speak out in front of groups”* (CM10). *“the industry’s largely ran by non-indigenous folk […]you see that any products being sold on the market is most likely coming from a non indigenous company”* (E6). *“Management of it [land] is often guided by government regulations that dictate how we have to do things”* (CM9).
Socioeconomic disadvantages—housing and food insecurity	*“[…] people that are on double incomes with kids who can’t afford fresh food”* (E4). *“I think food security is a real issue”* (CM11).
Lack of availability, accessibility and affordability of native foods	*“people don’t have the stock that I need, and I can’t find the seed”* (E9). *“Native ingredients are seasonal and also specific to each area […] for example, I know Kakadu plum only grows in the area of Kakadu, so the reason why there’s such a high price tag on things that are Kakadu plum infused is because they only come from this small area […] but also I think there are lot of Western or non-Aboriginal organisations are just putting the price tag up because they can and because they can profit off it”* (E1).
Intrinsic characteristics of native foods	*“Warrigal greens is a big one. It’s actually used as a salad and herb, but you have to like double boil, like boil it once and then again because of its effect on your kidneys”* (E9).
**Theme 3: Aboriginal aspirations for the food system**	
Reviving Aboriginal traditional knowledge and foods	*“I want more of that knowledge out there, whether it be in the schools, or whether it be in Woolworths, or Coles, or wherever, that knowledge needs to be out there, is it’s not just our knowledge, it’s knowledge for everyone. So, I’d like to see that as well”* (CM1). *“We need that knowledge to be ongoing”* (CM11). *“We want anyone to come and see our Aboriginal Australia, native Australia, the edibles, healing”* (CM3).
Food security and sovereignty integrating native foods	*“our native foods implemented into modern day foods”* (CM10). *“I think I want proper good food that is not damaging to the body […] I’d like to see more of our food out there”* (CM1) *“I think it would be great to include these [native foods] more into the food systems* (E6).
Harmonious Existence—A Sustainable and Healthy World	*“We have to plant in a sustainable way for the country in which we’re living in and breathing”* (CM11). *“We need to think about ways that we can do things better to be sustainable”* (CM9). *“I definitely want it to be a lot more environmentally friendly which I mean that’s what we just a circle answer. That it’s being more sustainable”* (E5).
**Theme 4: Reviving traditional knowledge and native foods**	
Values—Interconnectedness, Mother Earth, sharing and caring	*“Goodnight Daru in our language is Mother Earth, and she provides so we look after goodnight Daru because she looks after us”* (E3). *“Ohh you’re the crazy lady with all the fruit in the front yard that everyone steals? if people wanna pick a piece of fruit and eat it on the way to or from home or school, I’m happy for that to happen […] reciprocal relationships with gardens and community has to be thought of differently […] that everybody’s responsibility is to grow something and this notion of sharing food differently […] this Western way of thinking about “but they’re pinching your mandarins”. I don’t care. I planted them to be shared”* (CM11). *“growing food together, and that should be a cultural practice […] supplying each other with and becoming sustainable. As a family and as a community by growing our own food […] then eating it together to, you know, sharing […] I’m happy to share that information and as principal so that so then that person sharing information with me […]a place where we can go to and we can all sit together and share food and share ideas, conversations, stories”* (CM10). *I think that goes down to it, also to back to our traditional roots again, like when we talk about elders and knowledge and the way it’s passed on, no one is higher than anyone else. So it’s that reciprocal learning from each other and that sharing of knowledge […] let’s share this together and let’s learn together”* (CM9).
Principles (4 R’s)—Respect, Reciprocity, Relationships, and Responsibility	*“It’s gotta have something in it for them [community members], that could be extending cultural knowledge […] it could be that they can see a health benefit to them”* (E11). *“It is our ownership of Aboriginal communities, but it’s also having non Aboriginal people to work with us on to that traditional method of growing food practise and then connecting those communities as well through food […] Bringing people together, it’s empowering community “* (CM10). *“Let’s start listening to those who respect the earth”* (E2). *“Any knowledge is shared about native foods it needs to be in a culturally appropriate way and so I think that Aboriginal people can absolutely bring that and they can bring the knowledge […]we need to focus less on making every single native food available everywhere and really focus on areas that it can grow in”* (E1). *“there’s a lot of things that grow here that won’t grow out there and there’s a lot of things that grow out bush but they won’t grow here because the soil types, they are all different* (CM7). *“I try to only eat what’s around and seasonal”* (CM11).
Resources and Institutional support	*“[…] money needs to be put into communities where they are already doing this—where they already harvesting native foods, growing native foods because they might not realize the value of what they’re doing”* (E1). *“We have to make sure that government understands that this is going to be good policy. This is going to be election winning policy to do this […] We need engineers to start thinking about how we harvest these”* (E2). *“We need the educated people and we need the Elders, and we need to bridge the gap between the knowledge and learning systems” (E4).* *“it needs to be a big shift in that. To get a greater share of investment and research and development funds into native food and plants, propagation, and commercialisation”* (E7).
Sharing knowledge around native foods	*“informing those connections with families and communities about the traditional uses and the way I use it and get them to create that cultural practise”* (CM10). *“that’ll be good to have recipes”* (CM2). *“[…] you can have about 3 or 4 recipes to start”* (CM8). *“you talk about sustainable food production and sustainable food and sustainable like food systems and food security. This is how you plan it. This is one way of doing it”* (E11). *“our vision to start with was to grow native plants, so that we can educate our children that these were the plants that were growing and that these were the plants for headaches [and] this plant is a herb that you put it with your meat”* (CM7). *“we can have a tasting day… It’s a lovely idea”* (CM7). *“exposure […] trough our social media, through TV, TikTok and Facebook, we don’t have any of that basic cultural knowledge that we can that we already know it’s out there and that we can share”* (CM10). *“going to the community gardens, going to the schools or universities, the kids get so excited, and then they go back to the parents, and then all of a sudden they’re going out and buying this plant and growing it. They’ve got that connection with it and so that’s where I think it needs to start”* (E4).
Integrating native foods into the current food system	*“Yam daisies was a staple and some of them get fat and long and you cook them up and cook them on the coals”* (E11). *“saltbush…I pretty much you can get these like salty flavour in the bush and then they used it for making damper”* (CM2). *“I guess one of the most popular things is lemon Myrtle muffins”* (E11). *“for families who are renting apartments or renting […] we need to be able to show them that pots that can be moved or tubs around that are lightweight and can be moved, not growing really tall things”* (E11). *“We try and share those with the kids. We try to grow native grasses and native plants that are used so last week, not this week it was too hot at the high school, so they all sat there and weaved. They gathered native grasses and they made bowls. So we just so we try and teach the kids that that country as everything you need”* (E3). *“schools at the moment seem to buy a lot of the bush tucker. So they’ll be putting like kitchen gardens in and they want to incorporate native food into their kitchen garden”* (E10).
